# How Can Radiological Limitations in Atypical Clinical Submandibular Gland Küttner Tumor (IgG-4 Disease) Mimic an Atypical Occurrence of a Solid Salivary Gland Tumor?

**DOI:** 10.3390/diagnostics15243159

**Published:** 2025-12-11

**Authors:** Kamil Nelke, Klaudiusz Łuczak, Andreas Kouroumalis, Maciej Janeczek, Agata Małyszek, Stavroula Diamantopoulou, Evangelos Kalfarentzos, Christos Perisanidis, Maciej Dobrzyński, Piotr Kuropka

**Affiliations:** 1 Maxillo-Facial Surgery Ward, EMC Hospital, Pilczycka 144, 54-144 Wrocław, Poland; 2 Academy of Applied Sciences, Health Department, Academy of Silesius in Wałbrzych, Zamkowa 4 Street, 58-300 Wałbrzych, Poland; 3 Oral and Maxillofacial Surgery University Clinic, Dental School, National and Kapodistrian University of Athens, Evangelisms General Hospital, 11527 Athens, Greecemaciej.dobrzynski@umw.edu.pl (M.D.); 4 Department of Biostructure and Animal Physiology, Wrocław University of Environmental and Life Sciences, Kożuchowska 1, 51-631 Wrocław, Poland; agata.malyszek@upwr.edu.pl; 5 Department of Pediatric Dentistry and Preclinical Dentistry, Wrocław Medical University, Krakowska 26, 50-425 Wrocław, Poland; 6 Division of Histology and Embryology, Department of Biostructure and Animal Physiology, Wrocław University of Environmental and Life Sciences, Cypriana K. Norwida 25, 50-375 Wrocław, Poland; piotr.kuropka@upwr.edu.pl

**Keywords:** submandibular gland, cysts, tumor-like mass, solid mass, IgG4 disease

## Abstract

In the lateral neck area (LNA), the parotid glands and submandibular glands can be diagnosed with various lesions, especially cysts and tumors of different etiology. In the submandibular area, many lesions are related to salivary stones and some inflammations, causing a secondary gland enlargement. When no sialolithiasis is present, a close relation to other local inflammation causes, IgG4, or related chronic sclerosing disease should be estimated. Ultrasound evaluation seems to be sufficient to indicate any occurrence of salivary retention, inflammation, dilatation of ducts, and gland swelling, and to confirm the initial diagnosis of sialolithiasis or sialadenitis. Any possible tumor formation or tumor-like solid mass evaluation requires adequate computed tomography or magnetic resonance imaging. A very important question should be raised if clinical symptoms like neck asymmetry, submandibular swelling, and solid-mass formation always correspond with radiological as well as some worrisome oncological symptoms. On the other hand, when radiological imaging is insufficient or lacking, a fine needle biopsy would be useful. Problems arise when any signs of potential disease or other tumor-like lesions are inconclusive or indicate inflammation, and possible treatment options are limited. Secondly, when patient anamnesis and blood examination are normal, but a worrisome tumor-like appearance progresses in time, a serious question about improved diagnostics and scheduling for surgery should be raised. Not all cases of elevated serum IgG4 levels correspond with IgG4 lesions and the typical spectrum of those diseases, and therefore histopathological examination of excised lesion provides the scope of the following disease intensity. In the following interesting images, it is worth noticing that radiological, clinical, needle biopsy, and cytological examinations do not always correlate with each other, and in some cases, an open surgery should be considered to improve the diagnosis.

**Figure 1:**
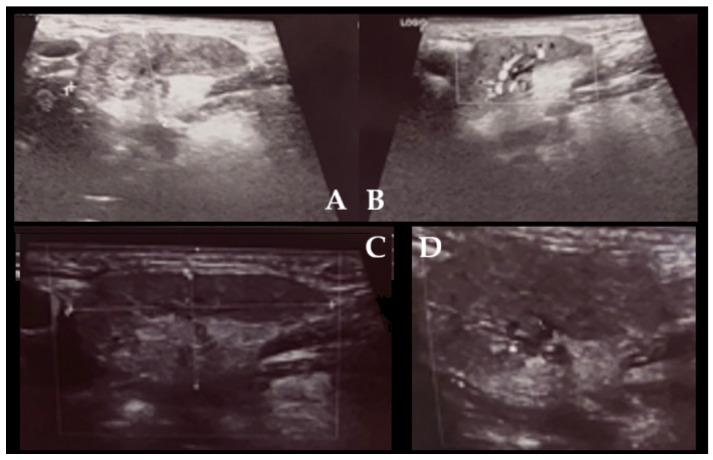
The submandibular gland (SG) is most commonly affected by sialolithiasis, sialadenitis, and related pathologies (30–80% of all cases). The occurrence and intensity of saliva retention caused by salivary stones within the gland and/or duct itself can have various stages and clinical significance. Those cases can be easily diagnosed with CT, CBCT, and USG. Mostly when pharmacological treatment and conservative endoscopic stone removal are not sufficient, then an open surgery consisting of submandibular duct surgery or salivary gland removal is performed. In some cases, a long-lasting chronic sialadenitis causes some pseudotumor changes in the SG that might occur. Gland asymmetry, sclerosing changes, and swelling of the SG might lead to a misdiagnosis with a para-neoplastic syndrome. The so-called Küttner tumor (KT, 1896) refers to a chronic sclerosing sialadenitis, which is a benign, non-neoplastic condition characterized by submandibular gland swelling, enlargement, and solid-like appearance. Rarely, other small glands may be affected as well. Typically, symptoms like pain, submandibular swelling induced by food intake, asymptomatic swelling and asymmetry, lack of salivary flow, pus secretion in the duct, as well as skin irritation, might be found. Normally, gland swelling and its solid appearance might suggest the occurrence of a benign or malignant lesion. Currently, KT is included as one of the IgG4 disease types but is not always related to IgG4 serum level elevation and typical histological changes. Other differential diagnoses should include other sclerosing diseases, sarcoidosis, Sjogren disease, or typical benign and malignant lesions of salivary gland origins [1,2]. Al-Deerawi et al. suggest that there is no consensus for treatment of KT, and it is mostly case dependent; however, the authors advise comparable ultrasound studies, while other authors suggest a biopsy or entire gland removal [1]. It is worth estimating that magnetic resonance (MRI) has more benefits than computed tomography, but both of them can influence the surgeon’s decision on the approach [1,2]. Not all KT might represent an increased IgG4 ratio in serum evaluation, which could represent various microscopic stages of the gland changes themselves, or not the full scope of IgG4 disease itself, based solely only on gland enlargement [1,2]. Initial ultrasound (USG) was not conclusive for any suspicious lesion; however, small chronic inflammation changes were noted, which were inconclusive for other typical lesions (**A**–**D**). A fine-needle biopsy is quite an important diagnostic tool. In the presented case, FNAB indicated some inflammation without any atypical cells. The submandibular asymmetry was present, the tumor-like mass was firm and tender, and the patient indicated its growth and some local sensitivity when shaving. On the other hand, additional diagnostic approaches include both improved endoscopy, full oral cavity, and pharynx evaluation with careful patient anamnesis to exclude any usage of potential esthetic dermal fillers and their migration or trauma in the area of neck asymmetry [3,4].

**Figure 2:**
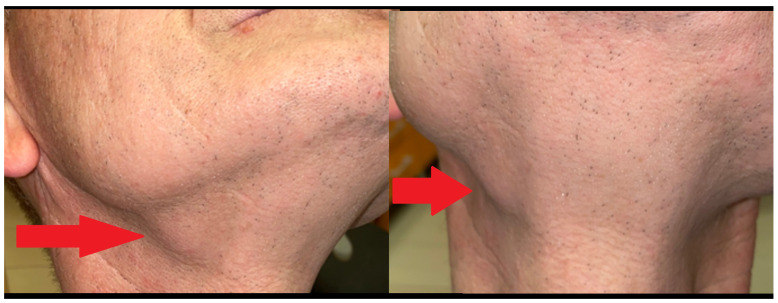
Most SG lesions have characteristic clinical and radiological patterns and can be easily diagnosed with basic steps. Some atypical asymmetrical, unilateral, and gland changes in its initial stages might lead to some troublesome diagnostics. A 60-year-old healthy patient complained about asymmetrical swelling in the right submandibular region and the presence of a solid-like lump (red arrow) with decreased mobility. Skin was healthy, and the saliva flow was proper on examination. Local lymph nodes were not enlarged and not palpable on examination. The patient denied the history of any cosmetic fillers, dental infections before the swelling, trauma, dental treatment, or other facial plastic surgery approaches. Sometimes, atypical cosmetic filler migration and accumulation might cause similar lesions to appear [3]. USG is a very accurate diagnostic tool for salivary glands. In cases of atypical swelling, any tumor-like mass evaluation should be differentiated from salivary stone-related sialolithiasis, saliva retention, and inflammation formation. On the other hand, because USG was inconclusive for a typical sialolithiasis, and neither was a solid tumor mass nor other mass within the gland, a decision was made to improve diagnostics by computed tomography (CT). Additional endoscopic nasal cavity and oro- and nasopharynx evaluation were performed, and possible teeth-related inflammation was also excluded. Based on a study by Su et al., a very good diagnosis can be made with the usage of polar-vessel color Doppler flow imaging (CDFI), for example, to confirm an adenoid cystic carcinoma or similar lesions of the submandibular gland [4,5,6]. On the other hand, major salivary glands, especially submandibular glands, might be diagnosed with chronic inflammatory or inflammatory-like processes, especially IgG4 or IgG4-related disease (IgG4/IgG4-RD). This process might have a typical inflammatory multi-organ condition or be just localized; however, the patient’s detailed examination and blood-work examination excluded this disease at the beginning. Because any silaolithiasis and IgG4 typical disease were excluded, as well as a solid tumor, a suspicion of a lesion of inflammatory origin, similar to old KT appearance, was raised. SG swelling, enlargement, asymmetry, and hardening suggested its cirrhosis and possibly fibrosis on palpation. Differential diagnosis of the other tumors with a solid-like appearance in the submandibular gland includes the following benign tumors: pleomorphic adenoma, Warthin’s tumor, myoepithelioma, oncocytoma, cystadenoma, lymphadenoma (LA), LEC (lymphoepithelial cysts, common for immunocompromised patients), sebaceous adenoma (SA), sialadenoma papilliferum, ductal papilloma (intraductal and inverted), canalicular adenoma, basal cell adenoma, and KT. It also included malignant lesions like mucoepidermoid carcinoma, adenoid cystic carcinoma, and other adenocarcinomas, including acinic cell carcinoma, salivary duct carcinoma, lymphoma, and metastatis (either from squamous cell carcinoma or other distant locations) [1,2,3,4,5,6]. Authors want to highlight the following unusual SG unilateral asymmetry and scope of possible diagnostic approaches.

**Figure 3:**
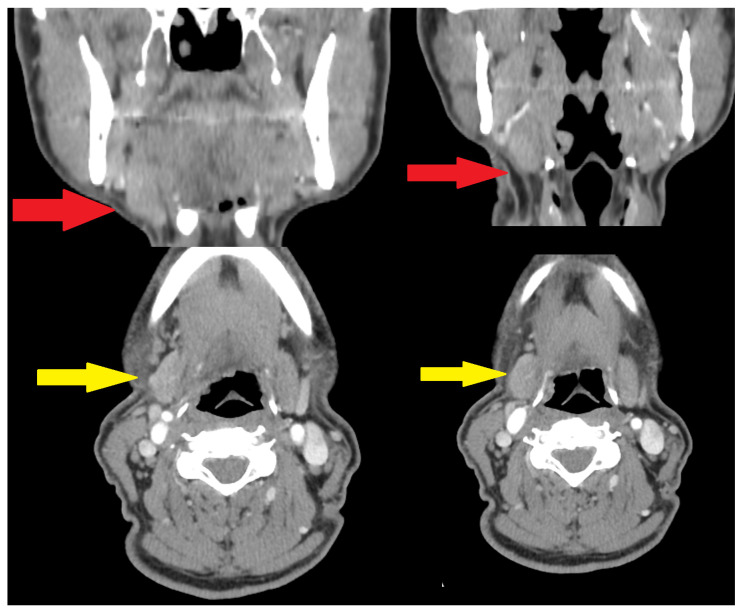
A computed tomography study suggested the presence of an atypically enlarged and asymmetrical right submandibular gland without any typical solid or cystic mass within the lesion (red and yellow arrows). The patient’s anamnesis excluded the presence of any submandibular gland swelling, inflammation, pus secretion, salivary stones, ductal stenosis, abscess formation, or any other atypical signs or symptoms. Because the patient suggested that the lesion had enlarged in size and in shape only recently, an atypical sclerosing lesion, similar to KT, was suspected. This atypical KT lesion itself is a rare condition, described by a German surgeon, Hermann Küttner, who reported an atypical unilateral submandibular gland swelling in 1896 [5,6,7]. Firstly, it was associated with chronic sclerosing sialadenitis present with swelling and submandibular gland asymmetry; however, nowadays, it is associated with the scope of IgG4 disease. This condition is quite often because of gland asymmetry, swelling, and the appearance of a solid-tumor-like mass, and other similar aspects are quite often misdiagnosed as a malignant tumor (the neoplasia-like syndrome, NLS). Therefore, when USG and FNAB are not accurate, and CT or MR have limitations in identifying potential lesions, an individual decision based on the patient case should be made. Currently, two possible options for this situation can be used, namely either “wait-and-see” and observe the lesion, or remove the lesion with the salivary gland to improve the diagnostics in histopathological studies. Lesions similar to malignancy-like tumors are challenging and require individual evaluation in each case. Typically, any IgG4/IgG4-RD lesion responds well to corticosteroid treatment, and if lesions decrease in size or limit themselves, then the response to therapy is good. Without any decrease in shape and size, a biopsy or excisional biopsy could be used, which is individual in each case. Other diseases differentiated with IgG4 include other diseases of IgG4 origin (Sjögren’s syndrome, Mikulicz’s disease, Castleman’s disease), which are found solely or overlapping with each other, and also other inflammatory diseases (tuberculosis, Crohn disease), as well as malignancies like cancers or lymphoma. An important aspect to note is that the serum IgG4 levels are not diagnostic on their own, especially when other conditions can cause their levels to be elevated. Because the lesion was firm and had with tendency to enlarge itself, a decision was made to remove the submandibular gland with the lesion itself [4,5,6,7,8].

**Figure 4:**
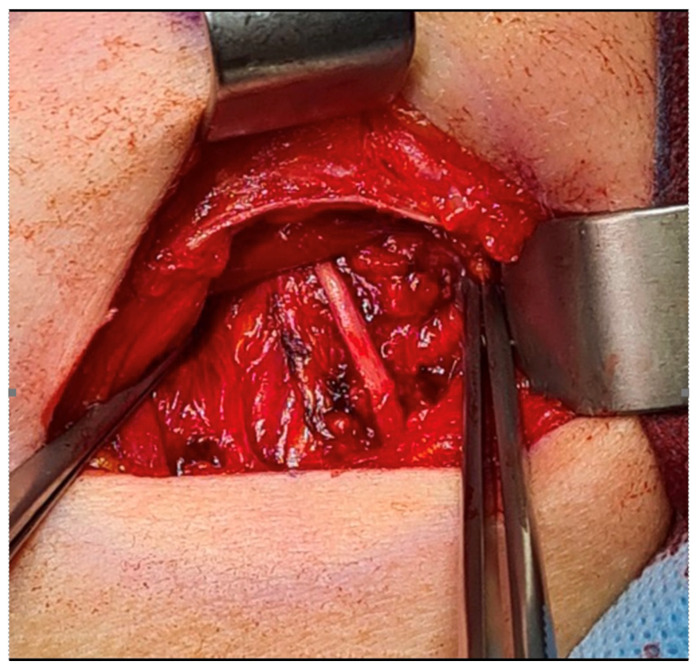
A surgery was scheduled under general anesthesia and oro-tracheal intubation. A typical approach from the submandibular area skin crease was used. After identification of the marginal mandibular branch of the facial nerve, the facial artery was retracted, and the entire submandibular gland was removed. The gland itself was hard, with a strange consistency and a solid-like appearance, without any adjacent enlarged lymph nodes in the area. Hemostasis was secured, a rubber drain was placed, and the entire wound was sutured in a layer-by-layer manner. Because the gland was solid in appearance with atypical macroscopic consistency, the decision for removal and its evaluation was sufficient in this case. It is worth remembering that both good, improved diagnostics with the usage of CT and/or MR can greatly increase the diagnostics, especially when combined with FNAB. This can easily estimate the type of lesions and the scope of necessary surgery. On the other hand, when the lesion is atypical, without any suitable diagnostics in use USG/FNAB and/or CT/MR, it is good to remove the entire lesion and ensure that a good specimen is studied. In those cases, if necessary, the scope of surgery can always be improved and radicalized locally if needed. Many authors suggest that CT and MR can greatly improve the scope of a surgical approach and the status of local lymph nodes or their diseases as well [5,6,7]. It is known that the submandibular gland has the possibility of expressing more malignant features than benign ones; therefore, a differentiation between the following lesions should be conducted: sialolithiasis, mucocele, epidermoid cysts, lymphoepithelial cysts (HIV+ patients), adenoid cystic carcinoma, mucoepidermoid carcinoma, Warthin tumors, pleomorphic adenomas, or others [7,8]. It is worthy to note that metastatic lesions from other different areas could be found in the neck area, especially in the glands and LNA: from breast, lung, thyroid, and head and neck squamous cell carcinomas (HNSCCs), as well as melanoma (both skin and mucosal once). It is found less commonly from renal clear cell carcinoma and hepatocellular carcinoma [8,9,10,11,12].

**Figure 5:**
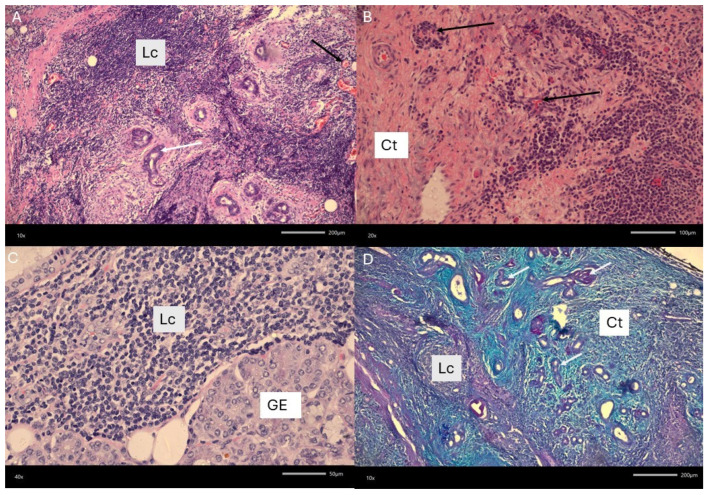
Initial histopathological evaluation included a chronic lymphocytic inflammatory process with local sclerosis, ductal atrophy, and chronic sialadenitis appearance, with the presence of a typical reactive lymph node in its proximity. The second evaluation was improved: To assess a precise picture of the changes, the material was fixed in 4% buffered formaldehyde with a pH of 7.2 for 24 h. Then, the material was dehydrated in an alcohol series and embedded in paraffin. Slides of 7 μm thick were stained with hematoxylin and eosin and the Azan-trichrome kit. Extensive inflammation and fibrosis were observed, with preservation of the overall lobular architecture of the submandibular gland. Acinar atrophy, resulting from chronic inflammatory processes and progressive fibrotic remodeling, led to the loss of normal acinar structures and the subsequent disruption of glandular function. Fibrotic changes are predominantly periductal, further compromising salivary flow and contributing to functional impairment. A swirling, cartwheel-like pattern of fibrous tissue known as storiform fibrosis is evident and represents a histopathological hallmark of IgG4-related disease. Additionally, obliterative phlebitis is present within the connective tissue, characterized by inflammatory infiltrates and fibrosis that occlude venous structures. Reactive lymphoid follicles are also noted, reflecting sustained immune activation within the glandular parenchyma. These follicles are composed of lymphocytes and plasma cells, consistent with an autoimmune etiology. The figure shows example pictures from the submandibular gland: infiltration of leukocytes (Lc) in glandular tissue; remaining collecting ducts (white arrows) and degradation of glandular epithelium (GE). Numerous obliterated vessels are indicated by the black arrow; collagen fibers of connective tissue are visible in place of the degraded secretory acini (Ct). Mag. (**A**,**D**) 100×; (**B**) 200×; (**C**) 400×. (**A**–**C**) Haematoxylin and eosin staining. (**D**) Azan trichrome staining.

**Figure 6:**
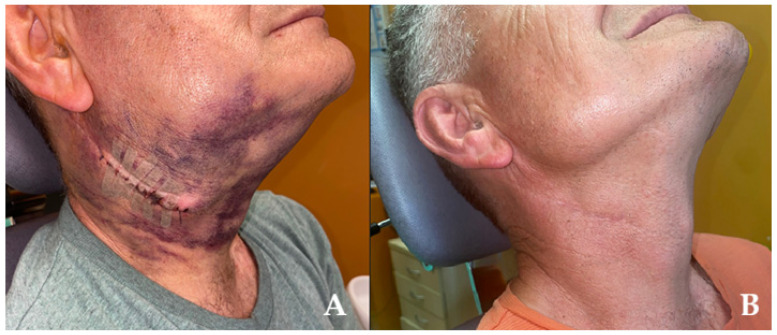
Postoperative period was complicated by massive hematoma formation and swelling without any bleeding (**A**). Cold-packings and compression therapy were applied with good overall success. Six months after surgery, the patient regained neck symmetry, shape, and all nerve functions were preserved; the scar is very esthetic, and an overall good final result was achieved (**B**). When improved blood serum evaluation for specific blood markers is not sufficient and radiological evaluation is limited, the FNAB should be helpful. In cases when cytology alone is inconclusive or not sufficient for final diagnosis, other steps should be considered. Lessons learnt from this case suggest that when atypical neck lesions appear, and when no explanation and diagnostics of the lesion are found, then an open biopsy grants more insight into the lesion, especially when its type can be evaluated during a preoperative histopathological evaluation. Some authors advise FNAB; however, as seen in this case, it also might have some limitations and might result in some inconclusive diagnoses. A study by Almulla et al. on 40 cases of salivary gland tumors indicated a 100% specificity and a low 23.08% sensitivity, with an overall accuracy of 73% [13]. On the other hand, a study by Lacerda-Oliveira et al. indicated that FNAB has a similar accuracy to incisional biopsy [14].

**Figure 7:**
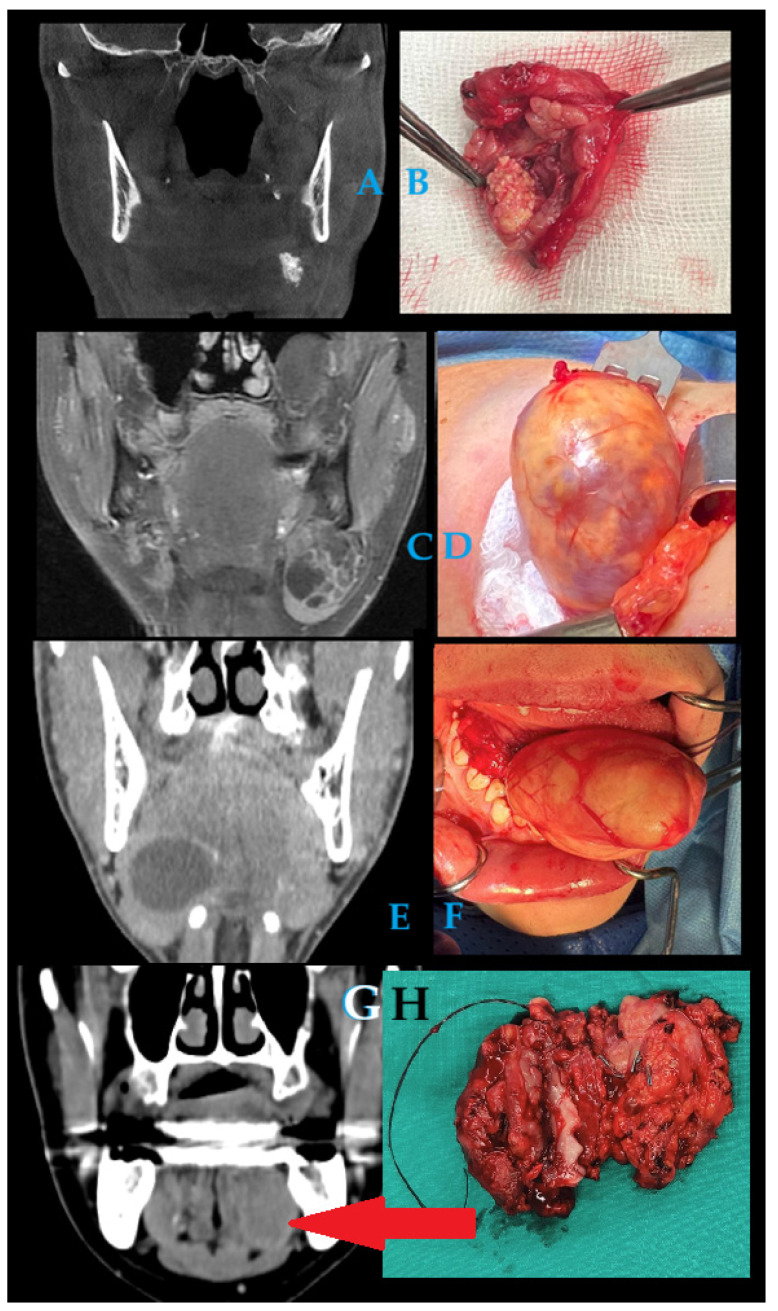
Most benign lesions in USG have visible borders, are well-defined, and have some characteristic cystic or cystic-like appearance. Each CT or MR improves radiological diagnostics. KT might have some atypical symptoms as well when it has a more solid than cystic appearance in USG, while in MRI, T1 is slightly hypointense; and in MRI, T2 might be slightly hyperintense [10,11,12,13,14,15]. During KT, levels of fibrosis might increase over time, which causes its appearance to be more related to a malignant lesion, rather than this condition itself. The levels of increased IgG4 serum values do not correspond with IgG4-type of lesion or KT alone, which could indicate that this lesion might have two different occurrence patterns [10,11,12,13,14,15]. FNAB and estimation of the levels of IgG4 serum could improve the diagnosis in some cases. Various forms of lesions in the oral cavity floor and the area of sublingual–submandibular glands might cause different forms of submandibular asymmetry, and structural tension, with some swelling responsible for a soft or solid-like appearance. Many typical salivary gland lesions have their characteristic radiological features, patterns, and can be distinguished in radiological studies alone. For comparison reasons, other radiological and clinical features of lesions within similar boundaries causing swelling and asymmetry are presented on images (**A**–**H**) located in the submandibular–sublingual gland or its direct proximity, namely (**A**,**B**) sialolithiasis of left submandibular gland (with visible salivary stone), (**C**,**D**) schwannoma of left submandibular gland and (**E**,**F**) dermoid cyst exceeding from right part of the oral floor to the right submandibular area, and (**G**,**H**) adenoid cystic carcinoma in its cystic-like form within the oral floor and sublingual gland (red arrow). All of the following lesions represent some inflammatory (**A**,**B**), benign (**C**–**F**), and malignant (**G**,**H**) characteristics, where their clinical and radiological features might easily identify their character. In many cases, radiology alone is quite precise in identifying some scope of lesions. Not all lesion types, boundaries, margins, and their behavior and type can be distinguished by radiological studies alone; therefore, improved diagnostics are mandatory. On the other hand, when some other worrisome clinical and radiological symptoms arise, a biopsy is more sufficient to later underline the most accurate surgical plan and prepare for the final scope of treatment. Regardless of the type of lesion having a radiolucent, radiopaque, or mixed appearance, when a CT is inconclusive, like in the presented case, and a biopsy/FNAB does not correspond with any reasonable diagnosis, an excisional biopsy is mandatory to gather an improved sample for histopathological study. Unilateral lesions in IgG4 are uncommon and might lead to misdiagnosis, suggesting atypical mass-forming fibrosing sialadenitis, and even a diffuse homogenous mass in CT with regular borders [16,17]. To summarize, any atypical growth in the SMG could suggest a malignant lesion where improved diagnostics are crucial, especially if FNAB and cytology might be insufficient or inconclusive. Presented KT could be easily misdiagnosed as any other inflammation. On the other hand, as indicated by Marcus et al., a typical KT related to IgG4-disease is not always caused by this disease, but it can also be caused typically by chronic sclerosing sialadenitis [15]. Adequate histopathological evaluation is crucial for the diagnosis of IgG4 disease, especially where other diagnostic measures are not sufficient.

## Data Availability

The raw data supporting the conclusions of this article will be made available by the authors on request.
